# Real-world effectiveness of denosumab compared to teriparatide in patients with severe osteoporosis

**DOI:** 10.3389/fendo.2026.1744054

**Published:** 2026-03-17

**Authors:** Maria Francesca Birtolo, Alberto Piasentier, Andreea Daniela Dutu, Walter Vena, Emanuela Morenghi, Stella Pigni, Lucrezia Maria Silvana Gentile, Alessandro Fanti, Valentina Vitale, Antonio Pucci, Gabriele Capo, Massimo Tomei, Carlo Brembilla, Maurizio Fornari, Andrea Laghi, Letterio Salvatore Politi, Antonio C. Bossi, Andrea Gerardo Antonio Lania, Gherardo Mazziotti

**Affiliations:** 1 Department of Biomedical Sciences, Humanitas University, Milan, Italy; 2 Endocrinology, Diabetology, Medical Andrology Unit, IRCCS Humanitas Research Hospital, Milan, Italy; 3 Radiotherapy and Radiosurgery Department, IRCCS Humanitas Research Hospital, Rozzano, Milan, Italy; 4 Endocrinology and Diabetology Unit, Humanitas Gavazzeni, Bergamo, Italy; 5 Biostatistics Unit, IRCCS Humanitas Research Hospital, Milan, Italy; 6 Neurosurgery Department, IRCCS Humanitas Research Hospital, Milan, Italy; 7 Radiology Department, IRCCS Humanitas Research Hospital, Milan, Italy

**Keywords:** denosumab, fractures, FRAX, severe osteoporosis, teriparatide, vertebroplasty

## Abstract

**Context:**

The potent inhibitory action on bone resorption and concomitant stimulatory effect on bone modeling make denosumab (DEN) as a possible alternative to anabolic drugs for treatment of severe osteoporosis.

**Objective:**

To compare the real-world effectiveness of DEN and teriparatide (TPTD) in patients with severe osteoporosis.

**Methods:**

This retrospective study included 357 patients (300 females, 57 males; mean age 70.2 ± 10.7 years) attending a referral center for osteoporosis in the period between July 2021 and June 2025. All the patients had indications for treatment with TPTD according to the national drug reimbursement criteria. However, 198 patients were treated with TPTD for 24 months, whereas 159 patients received DEN for contraindications to TPTD (110 cases), patient preference (39 cases) or side effects after few doses of the drug (10 cases). Patients were evaluated for clinical/morphometric VFs and non-vertebral fractures (NVFs) at baseline and after 24 months of treatment.

**Results:**

During the study period, 34 patients (9.5%) developed new VFs (clinical in 13 cases) and 7 patients (2.0%) experienced NVFs. The risk of new VFs was significantly associated with vertebroplasty procedure (odds ratio 2.409; p=0.037). Moreover, new VFs occurred less frequently in patients treated with TPTD as compared to DEN (12/198 vs. 22/159; p=0.013). In the multivariable analysis, the favorable effect of TPTD on risk of VFs was still significant after correction for vertebroplasty (odds ratio 0.39; confidence interval 95% 0.19-0.83; p=0.014). No significant difference in NVFs was found between DEN and TPTD (2/159 vs. 5/198; p=0.468).

**Conclusions:**

This real-world study shows that DEN might be less effective than TPTD in preventing VFs in patients with severe osteoporosis.

## Introduction

Prevention of fractures in clinical practice is carried out using several bone-active drugs with different mechanisms of action, with the choice guided by cost of drugs and the patient’s fracture risk profile. In patients with very-high risk of fractures, the guidelines recommend the use of anabolic drugs as first-line therapy (1, 2). Indeed, two randomized, controlled studies in postmenopausal women with severe osteoporosis have demonstrated the superior anti-fracture efficacy of skeletal anabolic agents, such as teriparatide (TPTD) a recombinant human parathyroid hormone analog (PTH 1-34), or romosozumab (a humanized monoclonal antibody sclerostin inhibitor), compared with anti-resorptive drugs (3, 4).

Denosumab (DEN), a fully humanized monoclonal antibody that inhibits RANK/RANKL/OPG signaling by competitively binding to RANKL, has been proven to be effective in patients with variable profile of fracture risk (5–7). Due to its dual effect of inhibiting osteoclast formation and function whilst stimulating bone modeling (8), DEN decreases bone turnover and induces a continuous increase in bone mineral density (BMD), with progressive decrease of fracture risk after several years of treatment (9, 10). Noteworthy, the efficacy of DEN was greater in subjects at moderate to high risk of fractures (11, 12). This evidence suggests that DEN might be an alternative for patients with severe osteoporosis who are candidates to TPTD (13), particularly those with contraindications to TPTD and older patients who may struggle to adhere to daily administration. However, to date, only few studies have compared head-to-head TPTD and DEN, and data were limited to BMD and bone structure without information on fractures (14–16).

This study aimed at comparing the real-word effectiveness of TPTD and DEN on risk of vertebral and non-vertebral fractures in women and men with severe osteoporosis.

## Materials and methods

### Study population

The retrospective study followed Strengthening the Reporting of Observational Studies in Epidemiology (STROBE) reporting guidelines (17). The inclusion criteria were: 1) indication to treatment with TPTD according to the current guidelines and national drug reimbursement, as defined by ≥ 3 VFs/hip fractures or one VF/hip fracture associated with either BMD T-score ≤-4.0 SD or long-standing (i.e., ≥ 1 year) glucocorticoid treatment or one VF/hip fracture occurring during first-line bisphosphonate therapy (18); 2) treatment with TPTD or DEN for 24 months; 3) availability of at least two spine images for vertebral morphometry longitudinally performed by the same machines during the study period; 4) written informed consent. Exclusion criteria were: 1) bone metastases; 2) non optimal adherence to bone-active-drugs during the study period. DEN was prescribed in place of TPTD in patients who had contraindications or refused treatment or showed side effects during the first 4 weeks of treatment. The adherence to TPTD was evaluated by the medication possession ratio (MPR) that was calculated as the percentage of days in which the subject had an osteoporosis medication available for use during the follow-up period. Patients were defined as adherent to TPTD if the MPR was ≥ 0.80 (19). In patients treated with DEN, adherence to therapy was defined when the drug was administered every 6 months ± 4 weeks (20). Vitamin D with or without calcium supplementation was given to all patients during study period. Calcium supplementation was given to subjects referring low intake of calcium with the diet and in those with secondary hyperparathyroidism.

In the period between July 2021 and June 2025, 357 patients (300 females, 57 males; mean age 70.2 ± 10.7 years) meet the inclusion and exclusion criteria and were retrospectively included in the study. For the purposes of the study, all participants were assessed at least at baseline and after 24 months of treatment with TPTD or DEN. The 24-month period was selected based on treatment reimbursement policies. Additional evaluations were carried out according to clinical judgement.

The primary endpoint of the study was the development of new VFs during DEN vs. TPTD therapy. As secondary endpoint, we explored the 1) occurrence of non-vertebral fractures (NVFs) and 2) determinants of fractures.

The study was approved by the Ethics Committee of IRCCS Humanitas Research Hospital (Study number: 4404; ID: 4536; Protocol code: 002), and the patients gave their informed consent to use the clinical data for research purposes.

### Clinical information

History of clinical VF and NVFs before and during treatment with TPTD or DEN was collected by clinical records. Moreover, in patients with clinical VFs, we collected information about eventual procedure of vertebroplasty. In patients older than 40, the fracture risk was assessed at the first clinical visit by the FRAX tool (FRAX^®^ tool) using the online calculator (www.shef.ac.uk/FRAX) with the information collected at the first visit (21).

All patients were evaluated by measurement of BMD at the lumbar spine, femoral neck, and total hip by using two DXA machines (Lunar GE or Hologic). BMD was expressed as T-score, comparing the results with those obtained in a gender-matched Caucasian population at the peak of bone mass. According to the WHO guidelines, a T-score ≤ − 2.5 SD at any of skeletal sites that we measured was defined as osteoporosis, whereas osteopenia was defined as a T-score between − 1 and − 2.5 SD (22). For the purposes of the study, only baseline BMD data were reported.

One hundred-seventy-three patients were evaluated at baseline for serum C-terminal telopeptide of type I collagen (CTX, i.e. a marker of bone resorption) (23). CTX was measured in blood samples on the morning after an overnight fasting, using the Elecsys β-CrossLaps/serum assay based on electrochemiluminescence technology and the COBAS e801 immunoanalyzer. The intra-individual coefficient of variation was 9.4% (4.1–27%) with a LSC of 27%. The reference ranges for males, pre- and post-menopausal women were 0.100-0.750, 0.136–0.689 and 0.177–1.015 ng/ml, respectively.

### Assessment of VFs

Vertebral fractures were evaluated using quantitative morphometry on conventional spine X-rays radiographs (24, 25). Six points were manually marked on each vertebral body to describe the vertebral shape. Anterior (Ha), middle (Hm), and posterior (Hp) vertebral heights were measured and height ratios (Ha/Hp, Ha/Hm, Hm/Hp) were calculated for each vertebra from T4 to L4. According to the quantitative morphometry method, the fractures were defined as mild, moderate, and severe based on height ratio decreases of 20–25%, 25–40%, and more than 40%, respectively (26). Morphometric assessment was performed at study entry and after 24 months of treatment. In case of symptoms suggestive for new clinical VF, an additional morphometric assessment was performed during the study period. New VFs were defined as either new fracture (from no VFs to any grade of VFs) or progression of pre-existing VFs (from mild to moderate/severe VFs; from moderate to severe VFs) between baseline and the follow-up. The evaluation of VFs was performed by two experienced physicians (G.M., A.P.). Discordant results were reviewed by a radiologist (L.S.P.) and the cases were resolved by consensus. Clinical VFs were defined by the presence of back pain associated with edema at magnetic resonance imaging.

### Statistical analysis

Continuous data were presented as mean ± standard deviation, unless otherwise stated. The Shapiro test was preliminarily employed to assess normality distribution of the data. Categorical data were presented as number and percentage. Normally distributed data were compared using t-test, whereas non-normally distributed parameters were compared by Mann-Whitney’s test. Unpaired frequencies were compared using the Chi-square test, with Fisher’s correction when appropriate. Factors associated with incident VFs were assessed by univariable logistic regression analysis. All risk factors significantly associated with new VFs in the univariable analysis and those with p-values below 0.1 were then submitted to multivariable logistic regression analyses. A P<0.05 was considered as significant. Statistical analysis was performed using SPSS version 25.0.

## Results

### Baseline characteristics

Among 357 enrolled patients, 302 (84.6%) were affected by primary osteoporosis, 43 (12.0%) by glucocorticoid-induced osteoporosis, 8 (2.2%) had osteoporosis associated to pituitary diseases and 4 (1.1%) had cancer treatment-induced bone loss. Densitometric diagnosis of osteoporosis was made in 227 patients (63.6%), whereas osteopenia or normal BMD were found in 102 (28.6%) and 28 (7.8%) patients, respectively. Multiple VFs were found in 336 patients (94.1%) and in 132 cases (37.0%) the VFs were clinical. Vertebroplasty was performed in 51 patients (14.3%) by 2 months prior to the study entry. NVFs were reported by 126 patients (35.3%). All 353 patients older than 40 who were evaluated by FRAX algorithm, were classified as at very-high risk of fractures.

One-hundred-thirty-nine patients (38.9%) were naïve whereas the remaining 218 patients had been already treated with bisphosphonates for at least one year prior to the study (167 treated with oral bisphosphonates, 51 with zoledronate). After the first visit, 198 patients (55.5%) were regularly treated with TPTD for 24 months, whereas 159 patients (44.5%) were treated with DEN in place of TPTD due to contraindications (n = 110), poor acceptance by the patients (n = 39) or early TPTD withdrawal due to side effects (n=10). The patients treated with DEN were significantly older and had more frequently primary osteoporosis than those treated with TPTD, without significant differences in sex, densitometric diagnosis of osteoporosis and fracture risk profile (**Table 1**).

**Table 1:** Baseline clinical and demographic data of 357 enrolled patients with severe osteoporosis, stratified according to the treatment modality (denosumab vs teriparatide) during the study period.

Variables	DEN	TPTD	p-values
N	159	198	
Age (year)	73.3 ± 8.9	67.7 ± 11.3	<0.001
Sex (F/M)	136/23	164/34	0.488
Naïve (%) Previous treatment with oral BPs Previous treatment with i.v. Zol	66 (41.5%) 67 (42.1%) 26 (16.4%)	73 (36.9%) 100 (50.5%) 25 (12.6%)	0.264
Type of OP (%)			
Primary OP GIOP OP-related to pituitary diseases CTIBL	143 (89.9%) 9 (5.7%) 3 (1.9%) 4 (2.5%)	159 (80.3%) 34 (17.2%) 5 (2.5%) 0	0.001
BMD categories (%)			
Osteoporosis Osteopenia Normal BMD	94 (59.1%) 52 (32.7%) 13 (8.2%)	133 (67.2%) 50 (25.2%) 15 (7.6%)	0.265
Multiple VFs (%)	153 (96.2%)	183 (92.4%)	0.129
Clinical VFs (%)	64 (40.3%)	68 (35.3%)	0.283
Vertebroplasty (%)	22 (13.8%)	29 (14.6%)	0.880
NVFs (%)	57 (35.8%)	69 (34.8%)	0.468
Serum CTX values (ng/ml)*	0.46 ± 0.45	0.35 ± 0.33	0.328

Continuous unpaired data were presented as mean and standard deviation, whereas categorical data were presented as number of cases and percentages. *, CTX was evaluated in 173 patients (65 under denosumab and 108 under teriparatide).

BPs, bisphosphonates; BMD, bone mineral density; CTIBL, cancer treatment-induced bone loss; CTX, C-terminal telopeptide of type I collagen; DEN, denosumab; F, female; GIOP, glucocorticoid-induced osteoporosis; i.v., intravenous; M, male; N, number; NVFs, non-vertebral fractures; OP, osteoporosis; TPTD, teriparatide; VFs, vertebral fractures; ZOL, zoledronate.

### Fractures during treatment with bone-active drugs

**Figure 1:**
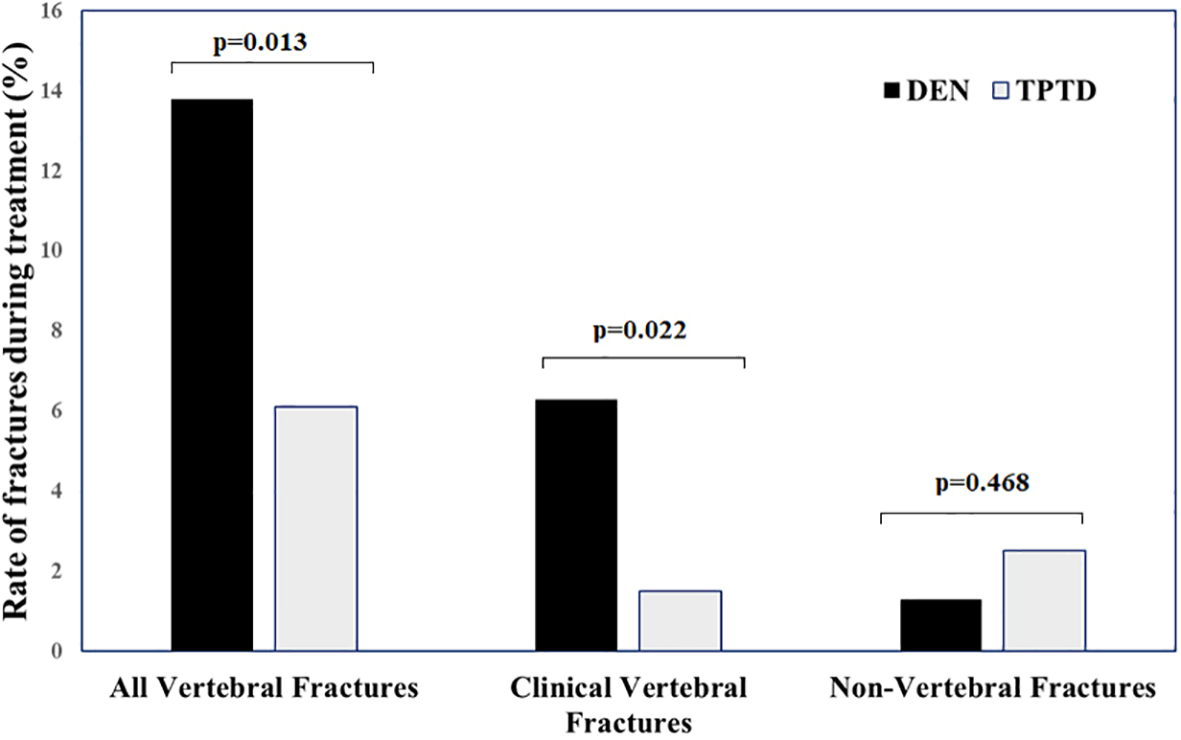
Rate of new vertebral and non-vertebral fractures during the study period in 357 patients with severe osteoporosis treated with teriparatide or denosumab.

**Figure 2:**
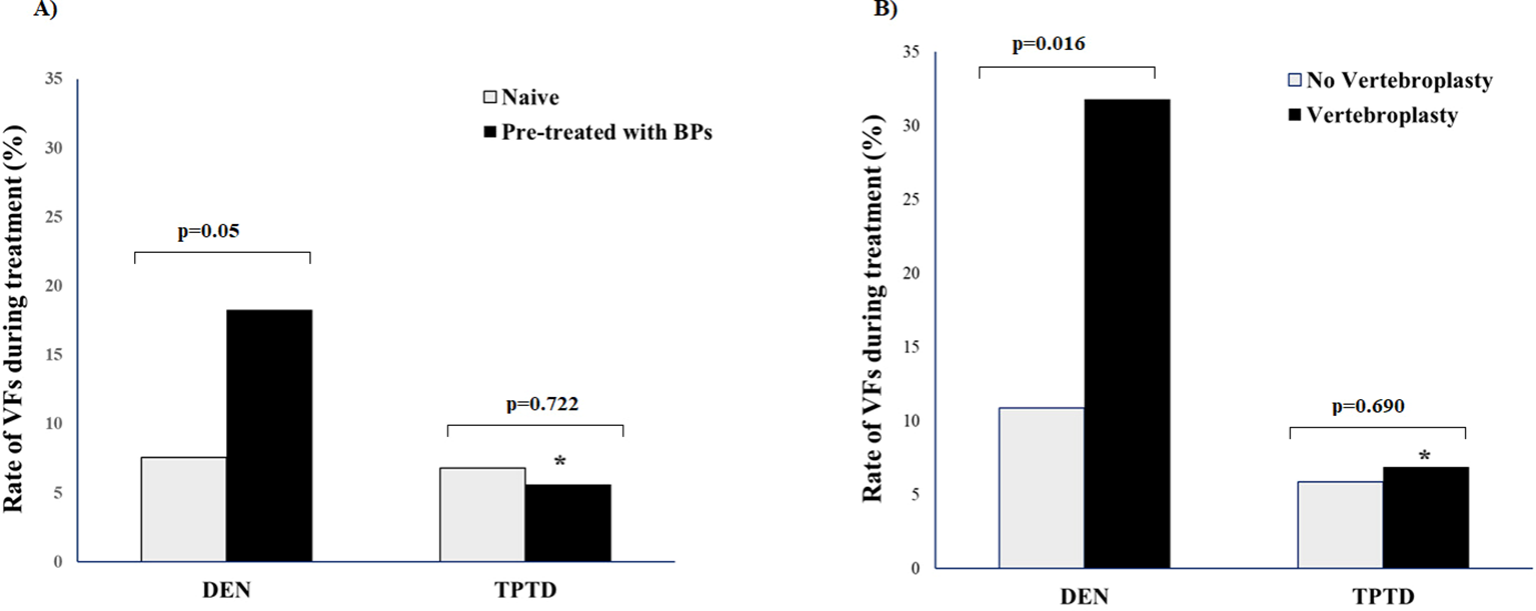
Rate of new vertebral fractures (VFs) during the study period in 357 patients with severe osteoporosis treated with teriparatide (TPTD) or denosumab (DEN) and stratified for previous treatment with bisphosphonates (BPs); **(A)** and vertebroplasty procedure **(B)**. *p<0.05 TPTD vs. DEN.

During the 24-month study period, 34 patients (9.5%) experienced new VFs, and 7 patients (2.0%) reported peripheral fractures. In 13 out of 34 patients developing new VFs, the fractures were clinical and 9 of them occurred during the first 12 months of follow-up. In the remaining 21 patients, VFs were morphometric and were diagnosed at the end of follow-up. The rate of new VFs was significantly lower in patients treated with TPTD than in those treated with DEN (all VFs, 12/198 vs. 22/159; p=0.013; clinical VFs 3/198 vs. 10/159; p=0.022), whereas no significant difference in NVFs was found between the two therapeutic arms (5/198 vs. 2/159; p=0.468) (**Figure 1**). Stratifying the patients for baseline characteristics, the rate of new VFs during DEN therapy was higher in patients pre-treated with bisphosphonates vs. naïve patients (**Figure 2a**) and in those undergoing vertebroplasty vs. those not treated with this procedure (**Figure 2b**). During TPTD therapy, the rate of new VFs did not significantly change in relationship with baseline characteristics and vertebroplasty procedure (**Figures 2a, b**). In naïve patients and in those not undergoing vertebroplasty no significant differences in new VF rate were found between DEN and TPTD (**Figure 2b**). In the univariable logistic analysis, new VFs were significantly associated with type of bone-active treatment (OR 0.402, C.I 95% 0.192-0.840; p=0.015) and vertebroplasty (OR 2.409; C.I. 95% 1.052-5.513; p=0.037) (**Table 2**). In the multivariable analysis, the association between low rate of VFs and TPTD therapy remained significant (OR 0.39, C.I. 95% 0.19-0.83; p=0.014) after correction for vertebroplasty procedure.

**Table 2:** Univariable logistic regression analyses evaluating the factors associated to new vertebral fractures during the study period in 357 patients with severe osteoporosis treated with teriparatide or denosumab for 24 months.

Variables	Odds ratio	C.I. 95%	p-values
Age	0.99	0.97-1.03	0.833
Male Sex	1.14	0.45-2-90	0.779
Naïve patients	0.63	0.29-1.35	0.234
Primary osteoporosis	3.17	0.61-16.44	0.169
DXA diagnosis of osteoporosis	1.07	0.50-2.29	0.857
Multiple VFs	2.18	0.28-16.76	0.455
Vertebroplasty	2.41	1.05-5.51	**0.037**
Treatment with TPTD	0.40	0.19-0.84	**0.015**
Baseline serum CTX values*	0.95	0.21-4.31	0.945

The values in bold refer to those statistically significant. *, CTX was evaluated in 173 patients (65 under denosumab and 108 under teriparatide).

C.I., confidence interval; CTX, C-terminal telopeptide of type I collagen; DXA, dual-energy x-ray absorptiometry; TPDT, teriparatide.

## Discussion

In this retrospective observational study involving patients with severe osteoporosis, treatment with TPTD was associated with lower rate of new VFs as compared to DEN, even when the drugs were used after bisphosphonates or after vertebroplasty procedure.

DEN is currently considered the most effective anti-resorptive drug for treating patients with osteoporosis, even when fracture risk is high (11, 12), for its mechanism on bone remodeling and modeling (8). Moreover, DEN might exert favorable extra-skeletal effects that could be potentially beneficial in the frail elderly population (27, 28). As a matter of fact, in real-world clinical practice DEN is often used in place of TPTD in patients with severe osteoporosis (13), but it’s still uncertain whether the two drugs might have comparable efficacy on risk of fractures since previous studies were focused on BMD and bone structures without providing information on fractures (14–16, 29). Our study confirmed that DEN is often used in clinical practice in place of TPTD (30, 31), and showed for the first time that DEN was less effective than TPTD in preventing VFs in patients with very high risk of fractures. These results reinforce the concept that anabolic therapy should be preferred in this clinical setting as first line-therapy. In patients with severe osteoporosis and absolute contraindications or irrevocable refusal of TPTD, effective alternative treatments, such as romosozumab, could be preferred over DEN (32, 33).

Our study provided some clinically interesting information about the performance of DEN and TPTD in specific subgroups of patients with severe osteoporosis. Noteworthy, TPTD seemed to be more effective than DEN even in patients previously treated with bisphosphonates, although there is a general consideration that TPTD may lose efficacy when used as a second-line treatment (34, 35). Over the last three decades, percutaneous vertebroplasty has emerged as a common treatment for clinical VFs possibly providing rapid symptomatic relief with consequent improvement in clinical outcomes (36). However, progression of VFs after vertebroplasty has been consistently reported, although it is still largely unclear whether and how the procedure might influence the skeletal outcome (37, 38). Indeed, treatment of osteoporosis with drugs able to reduce the imminent risk of fractures is expected to reduce the progression of VFs (6). Treatment with DEN was associated decreased incidence of new VFs after vertebroplasty procedure (39), although the number of events registered in the treated patients was higher than that already reported for patients not undergoing vertebroplasty (11, 12). Consistently, in our study the effectiveness of DEN resulted to be lower in patients treated with vertebroplasty vs. those not undergoing the surgical procedure. Differently, TPTD did not lose anti-fracture effectiveness in patients treated with vertebroplasty. This result was consistent with previous observations showing that TPTD was effective in preventing progression of VFs and controlling back pain (40–42).

While our study offers practical insights, some limitations should be recognized, mainly due to its observational nature. The study was observational and reflected the real-world clinical practice. Therefore, the allocation to the therapeutic arms was guided by clinical judgement and patient preference rather than random assignment, introducing selection bias. In fact, patients under DEN were older and showed more frequently primary osteoporosis than patients treated with TPTD. However, it is uncertain whether this selection bias might have influenced the final therapeutic outcomes, since patient’s age and type of osteoporosis were not associated with fracture risk when analyzed in the univariable logistic regression analysis. On the other hand, although statistical adjustments were applied, residual confounding cannot be fully excluded. Moreover, TPTD seems to be more effective than DEN notwithstanding the very-high risk of fractures related to chronic exposure to glucocorticoid therapy and the history of pituitary diseases (43, 44), that tended to be more frequent in patients treated with the former drug. These data confirm that TPTD is effective in reducing risk of fractures in patients with glucocorticoid-induced osteoporosis (45, 46), and provides a first evidence that the drug might be a therapeutic option in patients with pituitary diseases. Another limitation was inherent to the small number of patients, related to the monocentric nature of the study, which did not allow to perform a propensity score analysis, and reliable comparisons among different subgroups of patients with severe osteoporosis. Moreover, the study was not sufficiently powered to evaluate the effects of DEN and TPTD on risk of NVFs. Indeed, the number of NVFs appeared to be lower during DEN than TPTD, consistent with previous studies reporting better effects of DEN than TPTD on cortical bone (14–16, 29, 35, 47). Finally, the effect of timing and type of vertebroplasty on incident VFs occurrence were not assessed.

This study, within the limitations inherent in the retrospective design and the lack of treatment randomization, provides first evidence that DEN might be less effective than TPTD in preventing VFs in patients with severe osteoporosis, especially after vertebroplasty procedures. Future prospective larger studies are needed to confirm these observations.

## Data Availability

The raw data supporting the conclusions of this article will be made available by the authors, without undue reservation.
